# Validation of Early Increase in Complement Activation Marker sC5b-9 as a Predictive Biomarker for the Development of Thrombotic Microangiopathy After Stem Cell Transplantation

**DOI:** 10.3389/fmed.2020.569291

**Published:** 2020-10-06

**Authors:** Blanka Mezö, Orsolya Horváth, György Sinkovits, Nóra Veszeli, Gergely Kriván, Zoltán Prohászka

**Affiliations:** ^1^Research Laboratory, MTA-SE Research Group of Immunology and Hematology, Department of Internal Medicine and Hematology, Hungarian Academy of Sciences, Semmelweis University, Budapest, Hungary; ^2^Pediatric Hematology and Bone Marrow Transplantation Unit, Central Hospital of Southern Pest, National Institute of Hematology and Infectious Diseases, Budapest, Hungary

**Keywords:** hematopoietic stem cell transplantation, HSCT, transplant-associated thrombotic microangiopathy, TA-TMA, complement, pediatric, sC5b-9, thrombotic microangioapathy

## Abstract

Hematopoietic stem cell transplantation (HSCT)-associated thrombotic microangiopathy (TA-TMA) is a multifactorial complication. Complement dysregulation may play an important role in the pathogenesis of TA-TMA. Our previous observations suggested that early increase of soluble C5b-9 (sC5b-9), before the development of other complications, can predict the development of later TA-TMA. The present study aims to validate our earlier findings in an independent cohort enrolling 67 pediatric patients who underwent allogeneic HSCT during the study period (October 2015–January 2019). Five different TA-TMA diagnostic criteria were applied, and all important clinical and laboratory parameters of TA-TMA activity were registered. Complement pathway activities, components and sC5b-9 levels were systematically measured before transplantation and on days 28, 56, and 100 after HSCT. A strong and remarkable association still have been found between early increase of sC5b-9 (10 of 10 patients with TA-TMA vs. 27 of 57 without TA-TMA; *P* = 0.002) and the development of TA-TMA during 100 days post-transplantation. An increase in sC5b-9 concentration had 100% sensitivity and 53% specificity for TA-TMA in the cohort. All TA-TMA cases have been observed during cyclosporine immunosuppression, no TA-TMA was diagnosed during tacrolimus or mycophenolat mofetil therapy. In the majority of patients TA-TMA was mild and self-limiting, without any signs of organ damage. No additional complement parameters were closely associated with the development of TA-TMA. Early raise of the sC5b-9 activation marker was predictive for later development of TA-TMA throughout the whole study period. In patients with a marked increase, early and frequent monitoring of TA-TMA activity markers should be attempted, to facilitate subsequent therapy decisions in time. However, patients with TA-TMA were only identified during or after cyclosporine immunosuppression. Further studies enrolling higher number of patients are necessary to determine the role of immunosuppression in the pathogenesis of TA-TMA.

## Introduction

Transplant-associated thrombotic microangiopathy (TA-TMA) is a severe complication of hematopoietic stem cell transplantation (HSCT), characterized by microangiopathic hemolytic anemia, elevated serum lactate dehydrogenase level, thrombocytopenia, and multiorgan injury (1). Earlier studies reported incidence rates of TA-TMA between 0.5 and 63.6% (2), while more recent studies have shown the rate of 2.3 to 39% (1, 3). Although the exact pathophysiology is still unclear, endothelial damage appears to be central. Numerous risk factors have been identified including non-modifiable factors such as older age, number of prior transplantations, complement gene variations and CMV seropositivity of the recipient (4, 5). Multiple studies determined various transplant-associated risk factors like the use of HLA-mismatched donors and peripheral blood stem cells, infections, conditioning regimens and high-dose chemotherapy, graft-vs.-host disease (GVHD), and calcineurin inhibitors (6, 7). Recent research demonstrated that the presence of TA-TMA with concurrent infection or GVHD associates with worse survival than TA-TMA alone, furthermore patients who develop TA-TMA are also at increased risk of developing GVHD, infections and other complications (8, 9).

However, there is growing evidence that the overactivation of the complement is involved in the pathophysiology of TA-TMA. Jodele et al. reported that HSCT recipients with proteinuria and elevated soluble terminal complement complex sC5b-9 levels in blood at the time of TMA diagnosis were associated with very poor survival (10). In recent years, numerous studies have demonstrated the elevated level of sC5b-9 may aid in the diagnosis of TA-TMA, while patients with increased sC5b-9 levels are at higher risk of mortality, although not necessarily led to the development of TA-TMA (7, 11).

Recent studies suggest that elevated levels of other proteins and activity of the complement, like the activation product Ba, C3b and the total classical pathway activity (CH50) could serve as a marker of TA-TMA, supporting the role of the complement system in the pathogenesis of TA-TMA (11, 12).

These findings suggest that activation of the complement via the alternative pathway may lead to terminal pathway activation in TA-TMA and may help to identify patients who are at higher risk for the development of TA-TMA after HSCT.

In our previous pediatric cohort the early increase in level of sC5b-9 activation marker (from baseline until 28 days after HSCT) was proved to be predictive for the development of TA-TMA during the first 100 days post-transplantation with 100% sensitivity and 61% specificity. In the present study our aim was to validate the earlier observations in an independent, pediatric cohort, enrolling higher number of patients.

## Patients and Methods

The sample and data collection as well as the methods used for the detection and analysis of complement parameters were described with full details in our previously published paper (13).

### Patient Population, Sample, and Data Collection

**Figure 1:**
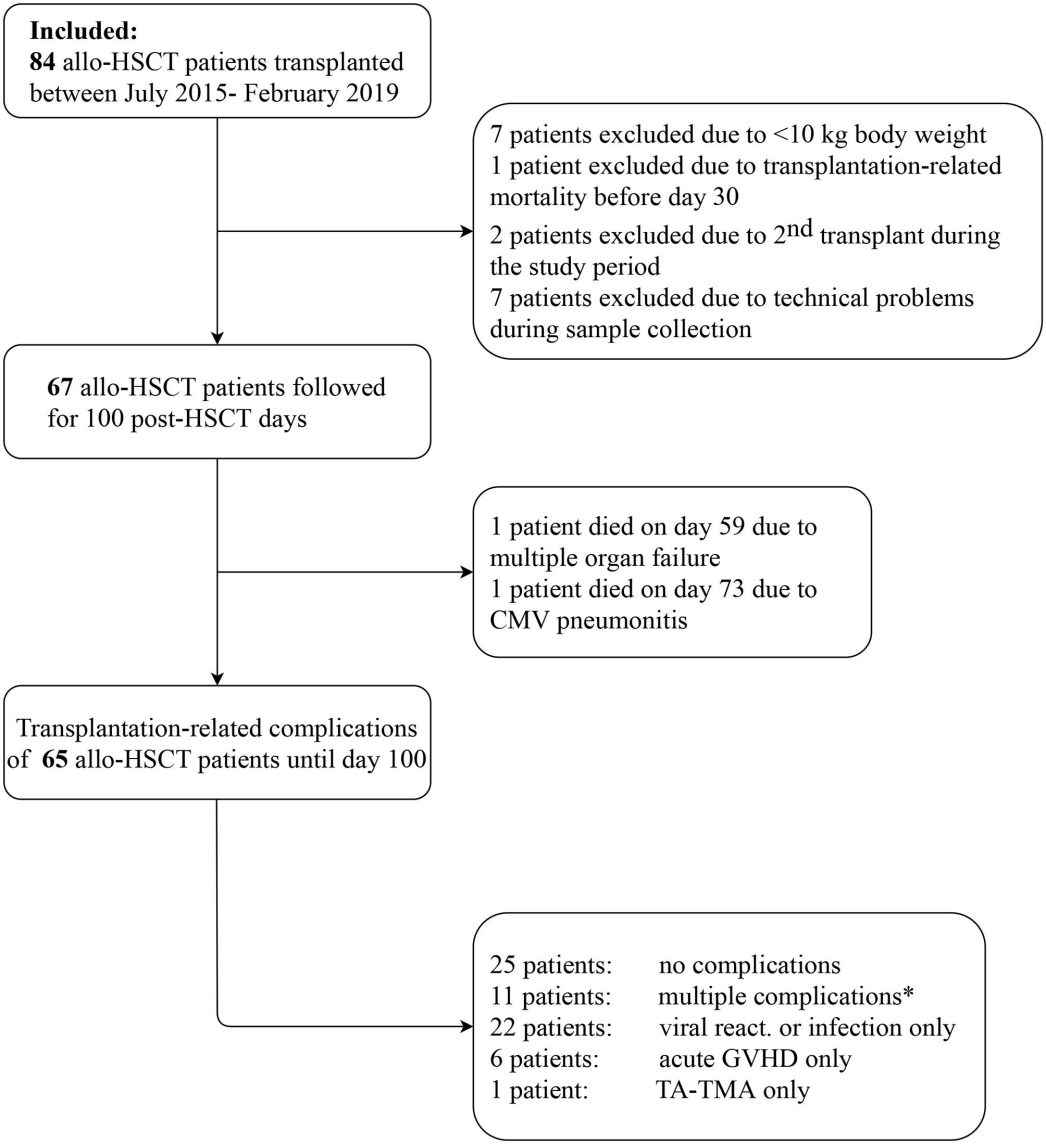
Study flow chart. ^*^The sequence of multiple complications, including acute GVHD (G), viral reactivation or new infection (V), and TA-TMA (T) were as follows: three patients G-T; three patients V-T; two patients G-V; 1-1 patient each: T-V, V-T-G, G-T-V.

All patients from July 2015 to February 2019 who underwent allogeneic HSCT at United St. István and St. László Hospital in Budapest were approached to participate in this study. In total, 67 subjects of the 84 fulfilled inclusion criteria and had adequate material for complement assessments. Individuals with weight below 10 kg (seven patients) and death <30 days after transplantation (one patient) were excluded from this prospective cohort. Seven patients were excluded due to technical problems during sample collection. Moreover, two patients underwent transplantation in both study periods, their complement data were included in the first cohort, and excluded from validation cohort. Detailed flow chart is presented on Figure 1. The study was conducted in accordance with the Declarations of Helsinki and approved by the institutional Ethics Committee on Human Research.

Sample and data collection were performed in the same way as in our previous paper. Briefly, samples (serum, EDTA-anticoagulated plasma, and sodium-citrate anticoagulated plasma) were collected at four time points: before transplantation, 28, 56, and 100 days after HSCT. Coombs test, hypertension, proteinuria, serum lactate dehydrogenase, haptoglobin and creatinine levels, new onset anemia, thrombocytopenia, and fragmentocytes as markers of TMA activity were also determined.

### Detection of Complement Parameters

Complement measurements were performed after the 100 days follow-up. All parameters of a subject were determined at a single time point. Serum concentration of complement C3 (reference range: 0.9–1.8 g/L) and C4 (reference range: 0.15–0.55 g/L) were determined by turbidimetry. CH50 (reference range: 48–103 CH50/ml) was determined by an in-house hemolytic-titration assay while an enzyme-linked immunosorbent assay was applied to measure C1q (reference range: 60–180 mg/L), terminal pathway activation complex sC5b-9 (reference range: 110–252 ng/ml) as well as factor H (FH) levels (reference range: 250–880 mg/L), and the activity of the lectin (Wieslab Comp AP320 kit, reference range: 35–130%, EuroDiagnostica, Malmo, Sweden) and alternative pathways (reference range: 70–125%). Factor I (FI) and factor B (FB) levels were measured by radial immunodiffusion (reference range: 70–130%). FRET-VWF73 substrate was used to detect the ADAMTS13 enzyme activity (reference range: 67–151%).

### Definition of Transplant-Related Complications

Glucksberg criteria was used to grade the severity of acute GVHD (14). Quantitative PCR assays were performed to detect viral reactivation or primary infection. During the first 100 days of the post-transplantation period, viral reactivation (CMV, EBV or adenovirus) have occurred in 32 cases. Acyclovir was started on the first day of conditioning therapy. As preemptive therapy rituximab, foscarnet, gancyclovir, and cidofovir were used.

For the determination of TA-TMA, five sets of diagnostic criteria were used (15–19) and the date of diagnosis was defined as the day when all of the TA-TMA diagnostic criteria introduced by Jodele et al. were fulfilled.

None of the patients who underwent complement evaluation received eculizumab treatment in the whole study period.

### Statistical Methods

Continuous data were expressed as the median and IQR (range from the 25th to the 75th percentile) and categorical variables as frequencies. Fisher exact tests were used to compare categorical data and Mann-Whitney, Wilcoxon or Friedman tests to compare continuous data.

Kaplan-Meier analysis was applied for calculation of TA-TMA even-free survival and Log-rank test was used for the comparison of survival curves. Two-sided *P*-value <0.05 was considered statistically significant. For analyzes GraphPad Prism 6.03 (GraphPad Software Inc., La Jolla, CA) and IBM SPSS Statistics 24 (IBM Corporation, Armonk, NY) were used.

## Results

### Baseline Characteristics

The study population included 67 pediatric patients undergoing allogeneic stem cell transplantation. The study flow chart is shown on Figure 1. The median age of the recipients at the time of HSCT was 8 years (range, 2.6–12.1 years), the majority of patients were male gender (55%). Malignant diseases were the primary indication of transplantation (55%), including acute lymphoblastic leukemia (46%), acute myeloid leukemia (22%) and juvenile myelomonocytic leukemia (11%) as the most common diseases. The non-malignant disorders (45%) involved were bone marrow failure syndromes, immunodeficiencies and metabolic diseases in 19 (63%), 8 (27%) and 3 (10%) patients, respectively. The conditioning regimen was myeloablative in 42 subjects (63%), the majority were treosulfan-based (52%) and busulfan was also used in 20 cases (48%). The most common graft source for HSCT was bone marrow (73%), particularly from matched unrelated donors. Patients were followed for 100 days after transplantation and transplantation related complications were monitored.

**Figure 2:**
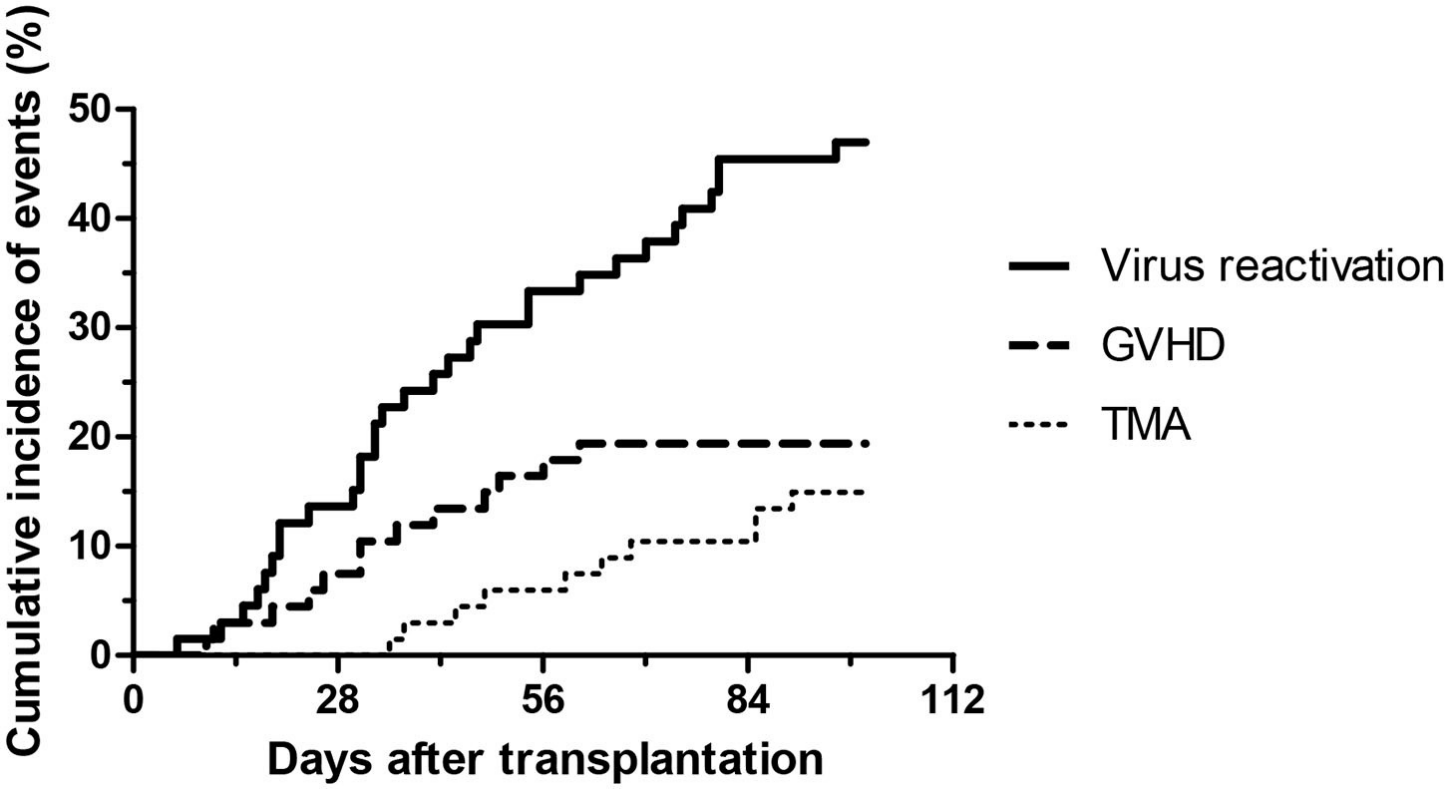
Development of virus reactivation, GVHD and thrombotic microangiopathy after HSCT from baseline to day 100.

During the first 100 days of the post-transplantation period acute GVHD was observed in 13 cases (19%), out of which 8 was graded as I-II, and 5 was graded as severe III-IV GVHD. Interestingly, out of the five severe acute GVHD cases, only two patient developed TA-TMA. Sixty-four of the 67 subjects received at least one of the calcineurin inhibitors as GVHD prophylaxis, including cyclosporine (*n* = 54) and tacrolimus (*n* = 13), furthermore mycofenolate mofetil was used in two cases, and one patient received no immunosuppression due to an identical twin donor. Sirolimus was used in two cases as GVHD prophylaxis or treatment. Furthermore, the most frequent transplant-related complication was virus reactivation which was detected in 33 patients (49%). Twenty-five patients had no complications, while most of the TA-TMA events were preceded by GVHD or viral reactivation (*n* = 9). Incidence of transplantation-related complications is shown on Figures 1, 2.

All components of the five various diagnostic systems for TA-TMA were monitored consecutively. Depending on the different diagnostic systems, 6 (9%)(BMT = 26), 5 (7%) (IWG = 27), 5 (7%)(Cho = 28), 7 (10%)(CoH = 29), and 10 (15%)(Jodele) of 67 subjects met the criteria for TA-TMA. All of the 10 patients identified by the criteria system of Jodele et al. fulfilled the requirements of at least three TA-TMA diagnostic systems.

Of 67 patients, 10 (15%) met the criteria for TA-TMA with the median occurrence of 62 days post-transplantation (range, 42–85 days). Characteristics of patient groups with or without TA-TMA are summarized in Table 1. No differences were found in relation to age, gender, previous transplantations, donor type, stem cell source, conditioning therapy, time of engraftment, and viral infections between the two groups. All TA-TMA cases have been observed during cyclosporine immunosuppression and no TA-TMA was diagnosed during tacrolimus (0 of 13 patients) or mycophenolat mofetil (0 of 2 patients) therapy. As a consequence of reduced toxicity conditioning regimen, in majority of patients TA-TMA was mild and self-limiting, without any signs of organ damage. Out of the 10 TA-TMA cases, three patients required intensive therapy due to organ damage during GVHD and TA-TMA treatment. In case of early signs of a developing TA-TMA, withdrawal or change of calcineurin inhibitors was the initial step to control the process in our clinical practice. Calcineurin inhibitor was discontinued or changed by the treating physicians if hypertension, posterior reversible encephalopathy syndrome, relapse, minimal residual disease or rapidly elevating LDH occurred. One patient received defibrotide therapy as TA-TMA treatment.

**Table 1:** Patient characteristics.

	**Patients without TA-TMA (*n* = 57)**	**Patients with TA-TMA (*n* = 10)**	***P*-value**
Age, years (median; IQR)	7.1 (2.5–11.8)	9.4 (6.7–14.3)	0.07
Male recipient sex	31 (54.4)	6 (60)	1
**Indication of transplantation**			
Malignancy	31 (54.39)	6 (60)	
Bone marrow failure	16 (28.1)	3 (30)	0.84
Other (immune deficiency or metabolic disease)	10 (17.5)	1 (10)	
**Recent transplantation**			
0	53 (93)	10 (100)	1
1	4 (7)	0	
**Donor type**			
Related identical or haploidentical	21 (36.8)	2 (20)	0.47
Unrelated	36 (63.2)	8 (80)	
**Stem cell source**			
Bone marrow	42 (73.7)	7 (70)	
PBCS	11 (19.3)	3 (30)	0.55
Cord blood	4 (7)	0	
**Conditioning therapy**			
Myeloablative	34 (59.6)	8 (80)	0.3
Reduced intensity	23 (40.4)	2 (20)	
**Engraftment, days**	20.5 (15–26)	26 (13–48)	0.41
**HSCT-related complications**			
Acute GVHD before day 100	8 (14.1)	5 (50)	0.019
GVHD before day 28	3 (5.3)	2 (20)	0.16
Viral infection before day 100	27 (46.6)	6 (60)	0.5
Viral infection before day 28	8 (14)	2 (20)	0.64
None of the 2	25 (43.9)	1 (10)	
GVHD or viral reactivation	30 (52.6)	7 (70)	0.012
GVHD and viral reactivation	2 (3.5)	2 (20)	
Relapse-related mortality	10 (17.5)	0	0.34
TRM	7 (12.3)	2 (20)	0.28

*Data presented are n (%) unless otherwise indicated*.

*PBSC, peripheral blood stem cell; IQR, interquartile range*.

However, individuals with acute GVHD during the first 100 days post-HSCT were at higher risk for the development of TA-TMA (*P* = 0.019). Although the difference in the incidence of viral infections was statistically not significant between the TA-TMA positive and negative groups (60 vs. 46.6%, *P* = 0.5), the co-occurrence of acute GVHD and virus reactivation was more frequently registered for subjects with TA-TMA.

### Complement Activation During the First 100 Days After HSCT

Our first goal was to identify the associations between various measures of complement profile and TA-TMA, by measuring the activities of the classical-, lectin- and alternative pathways, plasma/serum levels of C1q, C4, C3, FH, FI, FB, and sC5b-9, as well as the activity of ADAMTS13 during the first 100 days after transplantation (Table 2). The levels of complement proteins were considered to be elevated when an increase from baseline was observed.

**Table 2:** Complement pathway activities and activation product levels at different time points before and after transplantation.

**Parameter**	**Before HSCT**	**Day 28**	**Day 56**	**Day 100**	***P*-value**
**Classical pathway activity, CH50/ml** *Reference range: 48–103*					
TA-TMA (*n* = 10)	73 (56–95)	90 (74–110)	87 (77–106)	88 (72–120)	ns
No TA-TMA (*n* = 57)	70 (63–96)	77 (69–100)	79 (66–92)	72 (61–84)	
**Alternative pathway activity, %** *Reference range: 70–125*					
TA-TMA (*n* = 10)	87 (75–106)	105 (42–119)	92 (80–102)	106 (99–109)	ns
No TA-TMA (*n* = 57)	89 (69–107)	94 (72–109)	92 (63–102)	89 (70–99)	
**MBL pathway activity, %** *Reference range: 35–130*					
TA-TMA (*n* = 10)	117 (80–119)	115 (62–118)	105 (102–128)	122 (90–126)	ns
No TA-TMA (*n* = 57)	84 (18–145)	105 (20–150)	99 (12–162)	95 (21–120)	
**Complement C3, g/L** *Reference range: 0.9–1.8*					
TA-TMA (*n* = 10)	1.42 (1.26–1.44)	1.51 (0.95–1.79)	1.5 (1.28–1.96)	1.43 (1.33–1.73)	ns
No TA-TMA (*n* = 57)	1.35 (1.16–1.58)	1.37 (1.2–1.64)	1.31 (1.15–1.49)	1.25 (1.06–1.36)	
**Complement C4, g/L** *Reference range: 0.15–0.55*					
TA-TMA (*n* = 10)	0.38 (0.29–0.44)	0.38 (0.36–0.53)	0.45 (0.36–0.5)	0.5 (0.42–0.56)	0.009 for TA-TMA
No TA-TMA (*n* = 57)	0.36 (0.26–0.45)	0.39 (0.3–0.51)	0.35 (0.27–0.43)	0.33 (0.26–0.4)	
**FH, mg/L** *Reference range: 250-880*					
TA-TMA (*n* = 10)	690 (456–758)	564 (440–715)	479(369–897)	535 (419–761)	ns
No TA-TMA (*n* = 57)	448 (316–605)	468 (347–612)	449 (296–614)	444 (313.9–531)	
**FI, %** *Reference range: 70–130*					
TA-TMA (*n* = 10)	90 (83–102)	76 (73–83)	79 (70–89)	77 (72–94)	ns
No TA-TMA (*n* = 57)	88 (78–102)	86 (76–103)	88 (76–105)	81 (70–97)	
**FB, %** *Reference range: 70–130*					
TA-TMA (*n* = 10)	98 (89–102)	89 (85–102)	106 (67–122)	84 (74–98)	ns
No TA-TMA (*n* = 57)	90 (74–102)	84 (67–110)	82 (67–100)	85 (68–104)	
**ADAMTS13 activity, %** *Reference range: 67–151*					
TA-TMA (*n* = 10)	99 (81–116)	68 (60–96)	84 (65–117)	92 (58–117)	ns
No TA-TMA (*n* = 57)	91 (70–112)	78 (55–102)	93 (58–121)	96 (62–120)	
**sC5b-9, ng/ml** *Reference range: 110–252*					
TA-TMA (*n* = 10)	157 (128–202)	240 (219–376)	210 (161–272)	137 (126–148)	0.003 for TA-TMA
No TA-TMA (*n* = 57)	153 (113–220)	164 (110–201)	147 (120–189)	149 (108–201)	
**C1q, mg/L** *Reference range: 60–180*					
TA-TMA (*n* = 10)	90.9 (77.9-112)	101.6 (63-116.1)	102.6 (90-120)	93.1 (35-125.7)	ns
No TA-TMA (*n* = 57)	89.9 (73.2–105.5)	78.1 (68.5–95.5)	78.7 (64.2–91.1)	66.3 (50.7–84.8)	

*Data are presented as median (interquartile range). P-values were obtained with two-way analysis of variance*.

*ns, not significant*.

Among these parameters, complement terminal pathway activation complex sC5b-9 and C4 levels showed association with incident TA-TMA.

We observed a significant association between elevated sC5b-9 levels and TA-TMA development. Baseline sC5b-9 levels did not differ in patients with (interquartile range, 128–202 ng/ml), or without (interquartile range, 113–220 ng/ml) subsequent TA-TMA, furthermore the median levels remained within the reference range of the assay for both groups. However, on day 28, sC5b-9 levels of 4 of 10 subjects with TA-TMA exceeded 252 ng/ml, the upper limit of the reference range, while 27 recipients showed an increase in the group of patients without TA-TMA, and among them 6 reached the upper limit (*P* = 0.037). Peak sC5b-9 levels were detected on day 28 in both groups, however, concentrations were significantly higher in patients who later developed TA-TMA, than recipients without: 240 ng/ml (interquartile range, 219 to 376) vs. 160 (interquartile range, 110–201; *P* = 0.0008). Furthermore, this difference remained on day 56 (*P* = 0.001), while sC5b-9 levels measured before HSCT and on day 100 did not differ between them. Moreover, all patients with later development of TA-TMA showed and early increase (until day 28) of sC5b-9 (Table 3).

**Table 3:** Complications and activity markers with or without early elevation of sC5b-9 until day 28.

	**sC5b-9 elevation from baseline to day 28 (*n* = 37)**	**No change or decrease of sC5b-9 from baseline to day 28 (*n* = 29)**	***P*-value**
Engraftment day	23 (17–27)	18 (13–24.5)	0.022
TA-TMA before day 100	10 (27)	0 (0)	0.002
TA-TMA before day 28	3 (8.1)	0 (0)	
Acute GVHD before day 100	7 (18.9)	6 (20.7)	1
Acute GVHD before day 28	3 (8.1)	2 (6.9)	1
Viral reactivation before day 100	18 (48.6)	14 (48.3)	1
Viral reactivation before day 28	8 (21.6)	7 (24.1)	0.31
Fever on day 28	3 (8.1)	1 (3.5)	0.62
Bloodstream infection	11 (29.7)	8 (27.6)	1
Transplant failure before 100 days	3 (8.1)	1 (3.5)	0.62
**Diagnostic parameters for TA-TMA**			
Elevated LDH until day 28	5 (13.5)	5 (17.2)	0.74
Elevated LDH until day 100	24 (64.9)	13 (44.8)	0.14
New onset anemia until day 28	1 (2.7)	2 (6.9)	0.58
New onset anemia until day 100	18 (48.6)	13 (44.8)	0.8
New onset thrombocytopenia until day 28	0	0	
New onset thrombocytopenia until day 100	15 (40.5)	10 (34.5)	0.8
Low haptoglobin until day 28	10 (27)	8 (27.6)	1
Low haptoglobin until day 100	13 (35.1)	12 (41.4)	0.62
Fragmentocytes until day 28	13 (35.1)	12 (41.4)	0.62
Fragmentocytes until day 100	20 (54)	15 (51.7)	1
Proteinuria until day 28	6 (16.2)	5 (17.2)	1
Proteinuria until day 100	13 (35.1)	5 (17.2)	0.16
Hypertension until day 28	17 (45.9)	7 (24.1)	0.078
Hypertension until day 100	22 (59.5)	8 (27.6)	0.013
None of the seven	11 (29.7)	4 (13.8)	
One of the seven	12 (32.4)	13 (44.8)	0.28
At least two of the seven until day 28	14 (37.8)	12 (41.4)	

*Data presented are median (interquartile range) or n (%). P-values were obtained by Fisher's exact test*.

When absolute difference between day 28 and day 0 sC5b-9 values were calculated, ROC analysis identified 66 ng/ml increase, as the optimum cut-off point to identify TA-TMA patients with 90% sensitivity and 84% specificity (AUC: 0.869; 95% CI 0.776–0.963).

Changes in C4 levels after HSCT showed moderate, significant differences between patients with or without TA-TMA. Patients with TA-TMA showed increasing C4 on 56 and 100 days after HSCT, whereas in patients without TA-TMA C4 levels peaked on day 28. We have no formal explanation for this observation, but since multiple complications necessitating combined/long drug treatment coincided with development of TMA, cumulative effects drug- or inflammation related factors might be in the background of elevated C4. However, a direct relationship between elevated C4 levels and development of TMA cannot be excluded, requiring further studies to clear this association.

There was no significant association of TA-TMA with changes in classical, lectin and alternative pathway activities, and other complement parameters.

The relationship between the early increase of sC5b-9 and transplantation related complications and early laboratory or clinical markers of TA-TMA were also analyzed (Table 3). Based on the changes in sC5b-9 levels from baseline to day 28, two groups were defined, including patients with elevated, or with decreased or unchanging levels of sC5b-9.

**Figure 3:**
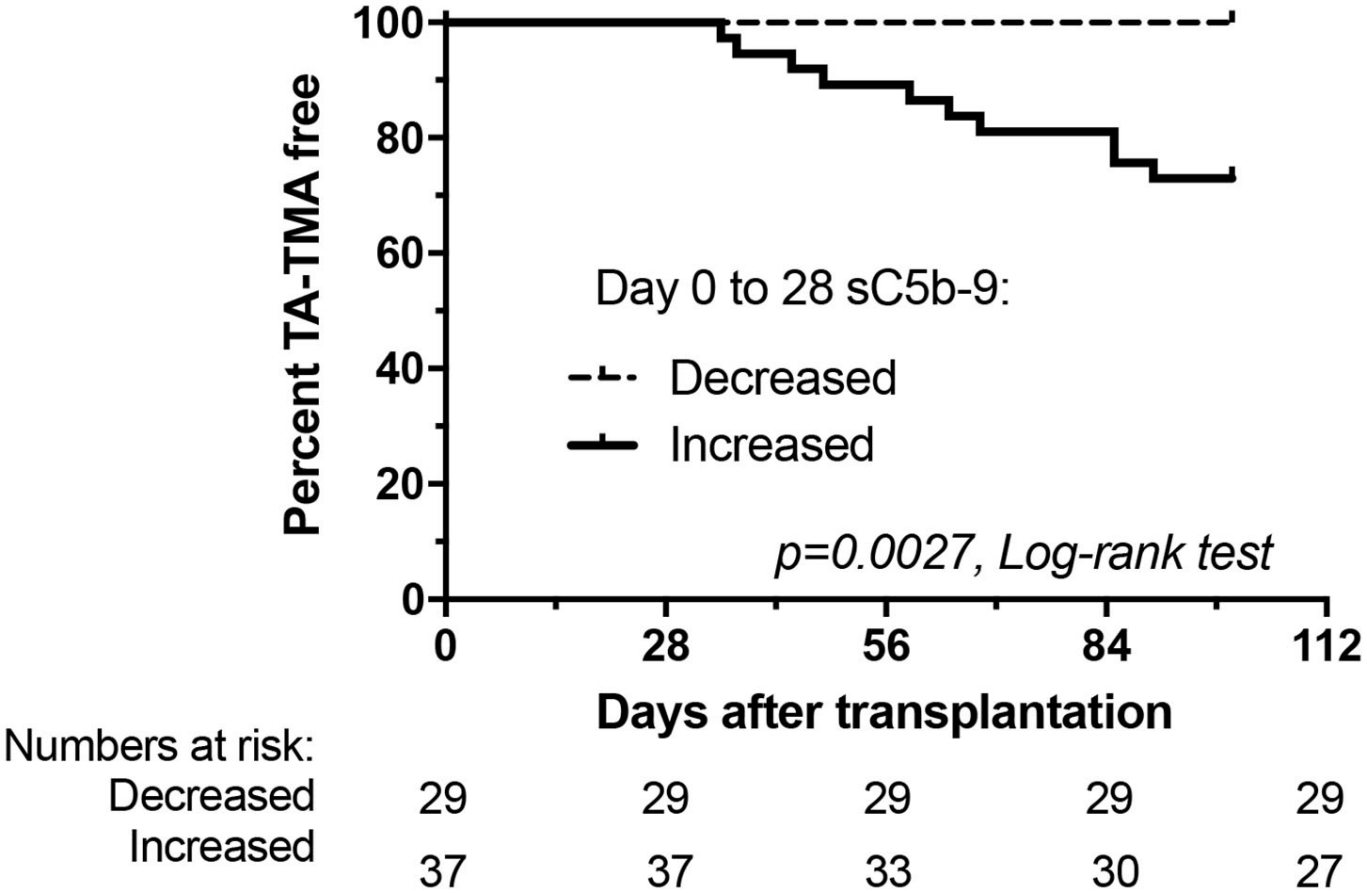
Development of thrombotic microangiopathy after HSCT in relation to changes of terminal pathway activation marker sC5b-9 from baseline to day 28. Kaplan-Meier plot showing percentage of TA-TMA event-free patients.

TA-TMA showed a remarkable association with early increase of terminal pathway activation (Figure 3). Ten patients of 37 with early increase in sC5b-9 levels developed later TA-TMA, while none of the 29 recipients without an elevation. Furthermore, the day of engraftment was delayed in patients with early increase in sC5b-9 levels. Acute GVHD, viral reactivation and/or infection, severe mucositis, fever, blood stream infection, transplant failure, elevated LDH, new onset anemia and thrombocytopenia showed no significant association with early increase of sC5b-9 levels (Table 3).

Finally, we analyzed if early increase of sC5b-9 levels could help in the clinical management of HSCT patients and calculated sensitivity and specificity values to predict development of TA-TMA. Early sC5b-9 elevation was a 100% sensitive predictive marker for the later development of TA-TMA (10 of 10 patients with TA-TMA had early elevation of sC5b-9; Figure 3), but it was less specific (10 of 37 patients with early sC5b-9 increase had TA-TMA events translating to 53% specificity). Positive predictive value of having early elevation in sC5b-9 for the development of TA-TMA was low, 27%, but the negative predictive value for the same (not having sC5b-9 elevation, and lack of development of TA-TMA) was 100%. Therefore, since 44% of our cohort had no early elevation of sC5b-9 levels we consider checking of sC5b-9 levels clinically useful to stratify patients for later development of TA-TMA.

## Discussion

Transplantation associated TMA is a frequent cause of organ damage, and is associated with increased morbidity and mortality after HSCT (20). Modifiable risk factors of TA-TMA are related to transplantation-associated factors like HLA- or minor ABO mismatched donor, use of PBSC, and lack of ATG- or presence of myeloablative conditioning regimen. In addition, several post transplantation events including infections, viral reactivation, acute graft vs. host disease and use of calcineurin inhibitors may also increase the risk of post-HSCT TMA (7). HSCT is planned process with strict protocols for biological sampling and monitoring of various organ functions, therefore, it is feasible to search for, and to identify predictive biomarkers of various post-TA-TMA complications. In our previous study we utilized the advantages of our institutional protocol and conducted an explorative, consecutive, comprehensive analysis of complement biomarkers to search for TA-TMA associated, predictive biomarkers. Early increase of terminal pathway activation marker was identified as a sensitive marker of later development of TA-TMA (13). To decrease the risk of a false positive conclusion that may be related to the small size of our previous observational study, we found it of utmost importance to validate our initial observations in an independent cohort, before making any firm conclusions based on the initial data.

Accordingly, the aim of this study was to enroll a new prospective cohort of pediatric patients undergoing HSCT, and to consecutively collect appropriate plasma and serum samples for complement analysis with relevant clinical information, in order to validate our previous results. Here we show, that early elevation of complement terminal pathway activation complex sC5b-9, is a predictive biomarker for the development of TA-TMA after HSCT (Figure 3 and Table 2). The current findings are fully supporting and validating our previous results, demonstrating 66 ng/ml increase, as the optimum cut-off point to identify TA-TMA patients with 90% sensitivity and 84% specificity (AUC: 0.869; 95% CI 0.776–0.963). The association between the early increase of sC5b-9 (binary variable) and later development of TA-TMA translates to remarkably high negative predictive value (100%) with 27% positive predictive value.

Most patients in our cohort had mild to moderate severity of TA-TMA (Tables 1, 3) and were managed by modification of immunosuppressive therapy, and by treatments for GVHD and infections. All TA-TMA cases have been observed during cyclosporine immunosuppression and no TA-TMA was diagnosed during tacrolimus or mycophenolat mofetil therapy. There was no patient in this cohort who required plasmapheresis or complement inhibitory therapy to manage TA-TMA. Accordingly, the mortality in this cohort was not associated with TA-TMA (Table 1). All, but one, of the 10 TA-TMA events were preceded by development of acute GVHD or viral reactivation, or both (Figures 1, 2). This observation is supporting the current “multiple hits” pathogenesis model of TA-TMA.

Currently, there are no universally accepted biomarkers for the prediction of TA-TMA, although platelet activation, neutrophil extracellular traps and complement activation are suggested to play key roles (21). The relationship between elevated sC5b-9 levels at the time of TA-TMA diagnosis and poor survival was reported a few years ago (10), which has been followed by several studies demonstrating the possible link between complement activation and TA-TMA, and facilitated subsequent therapeutic decisions and analysis to predict response to treatment (7, 22). Importantly, according to recent observations in a large pediatric cohort use of anti-C5 complement inhibitory drug seems to be an effective therapeutic strategy for high-risk TA-TMA patients after HSCT (22). In addition, Jodele and associates observed that subjects with higher complement activation marker sC5b-9 before initiation of anti-C5 treatment were less likely to respond and required more doses of the drug. Our results are in line of these observations and provide further support to complement activation markers, specifically terminal pathway activation marker sC5b-9, as clinically useful biomarkers to manage post HSCT TA-TMA patients.

In addition to sC5b-9 marker, our study identified development of hypertension and acute GVHD, as signs, and clinical factors associated with the development of TA-TMA. Additional risk factors for the development of TA-TMA after HSCT include conditioning agents and regimens (23). As a result of modifications in the institutional conditioning therapy (according to an international clinical study in patients with acute lymphoblastic leukemia a less toxic treosulphan based conditioning was used) and prophylactic immunosuppression protocol, the incidence of TA-TMA was lower in the current study (10/67) when compared to our original cohort (10/33; *P* = 0.05). As a consequence of reduced toxicity conditioning regimen, in majority of patients TA-TMA was mild and self-limiting, without any signs of organ damage and may contribute to smaller extent of sC5b-9 formation compared to sC5b-9 levels measured in our previous study. These observations indicate that the more aggressive or toxic conditioning regimens could lead increased TA-TMA incidence, although further, larger studies or pooled analysis, allowing multivariable analysis, would be necessary to identify and validate the exact composition and role of clinical risk factors for TA-TMA.

In conclusion, based on our original and current results we consider early elevation of sC5b-9, the terminal complement pathway activation marker, as a clinically useful predictive marker for the development of mild to moderate TA-TMA in pediatric HSCT patients. We suggest to include regular monitoring of complement biomarkers into additional studies and possibly also into institutional protocols. Studies on complement targeting drugs are very limited in this setting at this time, but interest is increasing after the first supportive clinical observations (22, 24). Accumulating data and evidence about the role and clinical utility of complement biomarkers, and advanced understanding of the exact role of complement activation in the post-transplantation setting may facilitate the development of biomarker-stratified therapy schemes, and improved management of the affected patients.

## Data Availability Statement

The raw data supporting the conclusions of this article will be made available by the authors, without undue reservation.

## Ethics Statement

The studies involving human participants were reviewed and approved by Scientific and Research Ethics Committee of the Medical Research Council (ETT TUKEB) in Budapest, Hungary. Written informed consent to participate in this study was provided by the participants' legal guardian/next of kin.

## Author Contributions

ZP and GK: study concept and design. BM, OH, GS, and NV: experimental procedures. OH and GK: acquisition of data. BM and ZP: critical writing of the manuscript. ZP and GK: study supervision. GK, OH, and ZP: acquisition of funding. All authors: data analysis, interpretation of data, and critical revision of the manuscript for important intellectual content.

## Conflict of Interest

The authors declare that the research was conducted in the absence of any commercial or financial relationships that could be construed as a potential conflict of interest.
